# 

**Published:** 1897-09

**Authors:** 


### Ohio State Dental Society.

The next meeting of this body will be held in Columbus, O., the first Tuesday in December, about ninety days hence. This time will quickly pass, and this fact should admonish all the membership who are to add anything to the interest of the occasion that each and every one should be astir.

A new era is upon us, beginning with the union of the American Dental Association and the Southern Dental Association and matters pertaining to this question, ought to come before every State Dental Society for consideration.

It is the desire and expectation that every such society will become a living auxilliary of the central organization—indeed a part of it. In addition to this a number of other very important subjects ought to have the wise consideration of our State societies.

Our dental law, together with the manner in which it is carried out, is by no means satisfactory to the profession, nor is it serving the purpose, in any good degree, for which it was obtained. Dental education is ever a living subject before our State societies.

It is to be hoped that the committee charged with the responsibility of making provision for this meeting will be actively astir in the near future, and that such arrangement will be made as to insure a large, interesting and profitable meeting.

Let every member and every one who intends to become a member, and every one who desires to be a visitor, mark upon his engagement book *December 6th to 10th, Ohio State Dental Society*, and make the writing so plain that it cannot be mistaken nor wiped out.

### THE DENTAL REGISTER,

**A MONTHLY MAGAZINE DEVOTED TO THE INTERESTS OF THE**

### DENTAL PROFESSION.

*Terms Reduced to* $2.00 *Per Year. Single Copies,* 25 *Cents.*

### EDITED BY PROF. J. TAFT, D.D.S.

—AND—

### Published by SAM'L A. CROCKER & CO., Ohio Dental and Surgical Depot.

The volume commences in January and closes in December.

The REGISTER being issued monthly is always fresh, therefore Indispensable to the practitioner who desires to keep posted up with the new inventions and improvements which are daily developed. Neither expense nor effort will be spared to give value and variety to its contents. Articles will be illustrated with well executed engravings whenever they will add to the interest or assist to explanation.

Its extensive circulation and frequency of issue make it one of the BEST DENTAL ADVERTISING MEDIUMS in the United States.

All subscriptions must begin with January or July.

Local or special notices, five lines or less, will be inserted for $3 each insertion; over five lines, 50 cts. per line.

All advertising bills payable in advance, or at furthest, after the issue of the first number containing the advertisement. All transient advertisements to be paid for in advance.

☞CONTRIBUTIONS SOLICITED.

Exclusively Editorial Communications should be addressed to the Editor.

The latest number of the REGISTER may be ordered with or without other goods [illegible] any time, and all letters should be addres[illegible]

